# ^18^FDG-PET/CT and molecular markers to predict response to neoadjuvant chemotherapy and outcome in HER2-negative advanced luminal breast cancers patients

**DOI:** 10.18632/oncotarget.24674

**Published:** 2018-03-27

**Authors:** Patricia de Cremoux, Lucie Biard, Brigitte Poirot, Philippe Bertheau, Luis Teixeira, Jacqueline Lehmann-Che, Fatiha A. Bouhidel, Pascal Merlet, Marc Espié, Matthieu Resche-Rigon, Christos Sotiriou, David Groheux

**Affiliations:** ^1^ Molecular Oncology Unit, Saint-Louis Hospital, Paris, France; ^2^ University Paris-Diderot, Sorbonne Paris Cité, INSERM/CNRS UMR944/7212, Paris, France; ^3^ Department of Biostatistics, Saint-Louis Hospital, Paris, France; ^4^ University Paris-Diderot, Sorbonne Paris Cité, INSERM UMR 1153 ECSTRA team, Paris, France; ^5^ Department of Pathology, Saint-Louis Hospital, Paris, France; ^6^ University Paris-Diderot, Sorbonne Paris Cité, INSERM UMR-S-1165, Paris, France; ^7^ Breast Diseases Unit, Saint-Louis Hospital, Paris, France; ^8^ Department of Nuclear Medicine, Saint-Louis Hospital, Paris, France; ^9^ Breast Cancer Translational Research Laboratory, Institut Jules Bordet, Université Libre de Bruxelles, Brussels, Belgium

**Keywords:** breast cancer, neoadjuvant chemotherapy, TP53 status, ^18^FDG-PET/CT, pathological complete response

## Abstract

**Background:**

The efficacy of neoadjuvant chemotherapy regimens in advanced luminal breast cancer patients is difficult to predict. Intrinsic properties of breast tumors, including altered gene expression profile and dynamic evaluation of metabolic properties of tumor cells using positron emission tomography/computed tomography (PET/CT) of tumor cells, have been identified to guide patient’s prognosis. The aim of this study is to determine if both analyses may improve the prediction of response to neoadjuvant chemotherapy in ER-positive / HER2-negative breast cancers (BCs) patients.

**Methods:**

We used metabolic PET parameters, at diagnosis and after two cycles of chemotherapy and proliferation gene expression profile on biopsy at diagnosis, in particular, the genomic grade index (GGI) analyzed by reverse transcription and quantitative polymerase chain reaction (RT-qPCR). The pathological response was the surrogate endpoint.

**Results:**

The change of FDG uptake between baseline PET and interim PET after 2 cycles of neoadjuvant chemotherapy (ΔSUVmax) was highly associated with pCR (p=0.008). We also observed an ability of P53 mutated status (p=0.042), in addition to histological grade (p=0. 0004), and PR expression (p=0.01) to predict pCR in ER-positive BCs, whereas no proliferation marker predicted pCR (P=0.39 for GGI). Finally, only ΔSUVmax was significantly associated with event free survival (p=0.047).

**Conclusions:**

Our results confirm the predictive and prognostic value of tumor ΔSUVmax in ER-positive /HER2-negative advanced BCs patients. These findings can be helpful to select high-risk patients within trials investigating novel treatment strategies.

## INTRODUCTION

Breast cancer (BC) is a heterogeneous disease with different phenotypes, each subtype having specific rates of response to therapy and specific prognoses [1, 2]. Among them, estrogen receptor (ER)-positive, HER2-negative luminal tumors have a more favorable clinical outcome than the other subtypes, i.e. basal-like and HER2-like [1, 2]. However, luminal A and B cancers have different prognosis and only a subset of them substantially will benefit from chemotherapy. Neoadjuvant chemotherapy (NAC) has been used for decades, to treat large or locally advanced breast tumors [3]. Recent research and clinical trials have shown strong correlation of breast cancer responses to neoadjuvant therapies with survival and prognosis, mainly in triple negative and HER2-overexpressing breast carcinoma [4]. Patients who achieve pathological complete response (pCR) to neoadjuvant therapy tend to have improved disease-free and overall survival compared with patients with residual invasive disease [4]. However, pCR rate still varies according to treatment regimen and intrinsic subtype of tumor and is achieved in only 5-10 % of luminal breast cancer [4, 5].

Classical clinico-pathological indicators of patients’ prognosis include tumor size, lymph node metastases, histopathological subtype, tumor grade, lymphovascular invasion, immuno-histochemical evaluation of hormone receptors, HER2 status and proliferation using Ki67 assessment. Nevertheless, the predictive value of these features in selecting the optimal therapeutic approach in NAC context is quite limited.

Different methods have been evaluated to improve the prediction of pCR and prognosis in patients treated with NAC. In particular, recently 2 markers showed promising results, the positron emission tomography/computed tomography (PET/CT) with 18F-fluorodesoxyglucose (FDG) [6–10] and gene expression profiling [4, 11–13].

In several studies, including ours, an association between early changes in the tumor FDG uptake (after one or two cycles of chemotherapy) and the final pathological response after completion of NAC have been observed [6, 7, 9, 10, 14]. However, the predictive and prognostic value of PET/CT has some limitations, especially in the ER-positive/HER2-negative breast cancer subgroup [15].

Recent developments of high-throughput methods such as microarrays and reverse transcription and polymerase chain reaction (RT-PCR) have allowed evaluation of biological markers, such as proliferation markers. In particular, the genomic grade index (GGI) was developed to improve BC grading and its prognostic value. It was associated to a better response to NAC including paclitaxel and fluorouracil, cyclophosphamide and doxorubicine in ER-positive and ER-negative BC, but associated with a worse prognosis [16]. It was initially designed with a panel of 97 genes differentially expressed in low and high histological grade breast tumors, and was consequently adapted for clinical use with a selection of 4 genes (CDC2, CDC20, KPNA2 and MYBL2), that may be analyzed in reverse transcription and quantitative polymerase chain reaction (RT- qPCR), with the same prognostic and predictive performances [12, 16, 17].

The aims of this study were to determine if metabolic PET parameters, assessed at baseline and after 2 cycles of chemotherapy, and/or gene expression profile, assessed at the time of initial diagnostic biopsy, along with clinical and pathological characteristics, may improve the prediction of response to NAC and of the outcome in ER-positive HER2-negative luminal breast cancers patients.

## RESULTS

### Patient’s and tumors characteristics

Patients and tumor characteristics of the 75 patients are described in Table 1. Most tumors were invasive ductal carcinoma, of large size (T3) and histological grade 2. More than half of the patients had lymph nodes involvement at clinical examination.

**Table 1:** Characteristics of the population of 75 ER-positive/HER2-negative breast cancers patients

	Number of patients (%)
**Patient’s age** ^#^	50 (44 to 60)
**Menopause**^†^	
Yes	23 (32)
No	49 (68)
Tumor Size^*^	
T0-T1	1 (1)
T2	26 (35)
T3	39 (52)
T4	9 (12)
Lymph node involvement^*^	
N0	33 (44)
N1	36 (48)
N2	5 (7)
N3	1 (1)
**Histological Type**	
Invasive ductal	67 (89)
Lobular	3 (4)
Other	5 (7)
Histological Grade^**^	
Grade-1	3 (4)
Grade-2	50 (67)
Grade-3	22 (29)
**Surgery**	
Lumpectomy	37 (49)
Mastectomy	38 (51)
**Pathologic Response rate**	
pCR	6 (8)
Non pCR	69 (92)

^#^ Median, interquartile range (IQR).

^†^Missing data: Menopause n=3.

^*^ Clinical classification before ^18^FDG-PET/CT according to the seventh edition of the AJCC Staging Manual.

^**^ Histological grade was assessed using the modified Scarff-Bloom-Richardson grading for invasive carcinoma.

pCR: Pathological Complete Response.

### Clinical parameters, molecular biomarkers, metabolic features and their correlation

In the series of 75 luminal breast cancers, a mean of 18% [7 to 38%] cells were stained by MIB-1 antibodies using KI67 and 46/75 (64%) samples were considered as proliferative tumors, by automated counting. ER or PR was overexpressed in all samples; mean level of expression was 80% [10 to 100%] in cells stained by anti-ERα antibodies. PR was overexpressed in 39 samples (52%); the mean level of expression was 10% [0 to 100%] in cells that were stained by anti-PR antibodies. Twenty samples presented a non-functional TP53 status (27%). The mean levels of Ki67, CDC2, CDC20, KPNA2 and MYBL2 gene expression were 288, 70, 186, 215, and 146 respectively (arbitrary units) (Table 2).

**Table 2:** Association between clinical, pathological, molecular and metabolic markers and pCR in the population of ER-positive, HER2-negative breast cancers (categorical variables as count (%) and continuous variables as median(interquartile range))

	N patients	No pCR	pCR	P^†^
**Total number of patients**	75	69	6	
**Tumor size**				0.66
T0-T1-T2	27 (36%)	24 (35%)	3 (50%)	
T3-T4	48 (64%)	45 (65%)	3 (50%)	
**Histological Grade**				
Grade-1-2	53 (71%)	53 (77%)	0 (0%)	**0.0004**
Grade-3	22 (29%)	16 (23%)	6 (100%)	
**Biological markers**				
**ER positivity n(%)**	74 (99%)	68 (99%)	6 (100%)	1
**PR positivity n (%)**	39 (52%)	39 (57%)	0 (0%)	**0.01**
**P53 status**				
Mutated	20 (27%)	16 (24%)	4 (67%)	**0.042**
Wild type	54 (73%)	48 (76%)	2 (33%)	
**Proliferation markers**				
**Ki67 (IHC)**	18 (7 to 38)	18 (6 to 37)	26 (15 to 50)	0.34
**Ki67 (RT-qPCR)**	288 (12 to 2131)^*^	304 (12 to 2131)^*^	241 (26 to 420)^*^	0.31
**CDC2**	70 (8 to 373)^*^	70 (8 to 373)^*^	67 (17 to 154)^*^	0.61
**CDC20**	186 (12 to 899)^*^	200 (12 to 899)^*^	120 (49 to 576)^*^	0.33
**KPNA2**	215 (17 to 6723)^*^	227 (20 to 6723)^*^	194 (17 to 248)^*^	0.15
**MYBL2**	146 (15 to 4131)^*^	146 (15 to 4131)^*^	150 (23 to 555)^*^	0.79
**GGI**	153 (104 to 324)^*^	153 (106 to 324)^*^	143 (57 to 174)^*^	0.39
**Metabolic markers**				
SUV max tumoral PET1	6 (4 to 8)	6 (4 to 8)	7 (6 to 10)	0.34
SUV max tumoral PET2	4 (2 to 5)	4 (2 to 5)	3 (2 to 5)	0.35
ΔSUV max tumoral (%)	34 (22 to 50)	33 (17 to 48)	62 (50 to 73)	**0.008**

^†^P=P-value for Fisher’s exact test (discrete variables) and Wilcoxon’s rank sum test (continuous variables); values in bold font correspond to values inferior to significant threshold 0.05, indicating significant association.

^*^Arbitrary Units.

Median tumor SUVmax of the 75 primary breast tumors was 6 (IQR: 4 to 8) at baseline and 4 (2 to 5) after the second cycle de chemotherapy. The median ΔSUVmax after 2 cycles was 34% (22% to 50%) (Table 2). No association was evidenced between clinical parameters and SUVmax, particularly, menopausal status was not associated with baseline SUVmax (p=0.54)

As expected, a high correlation was found between the expression level of GGI and its components (CDC2, CDC20, KPNA2 and MYBL2) (for example, relation between GGI and CDC20: rho=0.92, 95%CI: 0.86-0.95; Table 3). A positive correlation was also observed within the Ki67 measured by RT-qPCR (Ki67 mRNA) and GGI genes. On the opposite, no strong correlation was observed between the Ki67 measured by IHC (Ki67 automated) and Ki67 mRNA and between the Ki67 automated and GGI genes. No strong correlation was found between the baseline tumor SUVmax or histological grade and the molecular biomarkers (KI67 automated, Ki67 mRNA, GGI and its components) (for example, correlation between SUVmax and GGI: rho=0.28, 95%CI: 0.003-0.51; Table 3).

**Table 3:** Correlations between tumor SUVmax and proliferation-related parameters (pairwise Spearman rank correlation coefficient and confidence intervals)

	SUVmax	Histological grading	Ki67 automated	Ki67 mRNA	CDC2	CDC20	KPNA2	MYBL2
Histological grading	0.3 (0.08;0.51)							
Ki67 automated	0.23 (-0.01;0.47)	0.32 (0.12;0.53)						
Ki67 mRNA	0.14 (-0.11;0.38)	0.25 (0.02;0.49)	0.43 (0.22;0.63)					
CDC2	0.15 (-0.1;0.39)	0.33 (0.12;0.54)	0.31 (0.09;0.53)	0.83 (0.71;0.91)				
CDC20	0.32 (0.07;0.54)	0.25 (0;0.48)	0.33 (0.1;0.54)	0.85 (0.75;0.91)	0.74 (0.58;0.84)			
KPNA2	0.18 (-0.06;0.4)	0.2 (-0.04;0.44)	0.29 (0.07;0.49)	0.77 (0.62;0.87)	0.76 (0.62;0.85)	0.71 (0.54;0.83)		
MYBL2	0.27 (0.04;0.49)	0.35 (0.13;0.55)	0.34 (0.13;0.55)	0.76 (0.63;0.85)	0.8 (0.69;0.88)	0.8 (0.68;0.86)	0.72 (0.58;0.82)	
GGI	0.28 (0.03;0.51)	0.3 (0.07;0.53)	0.35 (0.11;0.55)	0.86 (0.76;0.92)	0.86 (0.77;0.92)	0.92 (0.86;0.95)	0.86 (0.75;0.93)	0.92 (0.86;0.94)

Scale for Spearman rank correlation coefficient absolute value. A zero value corresponds to the absence of correlation and an absolute value of 1 to perfect correlation. A positive correlation coefficient indicates positive correlation, i.e. higher values of variable A correlate to higher values of variable B. A negative correlation coefficient indicates inverse correlation, i.e. higher values of variable A correlate to lower values of variable B.

[0 - 0.20)
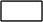
[0.20 - 0.40)
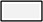
[0.40 - 0.60)
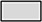
[0.60 - 0.80)
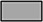
[0.80 - 1]
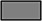

### Predictive markers of pCR

Clinical parameters were not associated with pCR, including menopausal status (p=0.66; Table 2).

Only 6 (8%) patients reached pCR after NAC. The baseline size of tumors with subsequent pCR was similar to that of tumors without pCR, with a mean of 4.6 cm (range 2-6 cm) vs. 5 cm (range 3.5 - 15 cm), respectively (p= 0.41). The level of ER expression by IHC was not different in pCR vs. non-pCR patients (100% and 99% positive samples respectively, p=1). On the opposite, the level of PR expression by IHC was different in pCR vs. non-pCR patients (0% and 57% positive samples respectively, p=0.01). Tumor grade was also predictive of pCR (p=0.0004; Table 2); all the tumors that reached pCR were grade 3 tumors. Contrarily to the histological grade, the GGI and its components CDC2, CDC20, KPNA2 and MYBL2 were not associated with pCR. Neither were the expression of Ki67 (Ki67 automated) and Ki67 mRNA. However, TP53 status was predictive of pCR. Tumors with mutated TP53 responded better to chemotherapy than tumors with functional TP53 (67% vs 24%, respectively; p=0.042).

Regarding PET parameters, while the tumor SUV_max_ measured at baseline and after 2 cycles of chemotherapy were not predictive of PCR (p=0.34 and 0.35, respectively), the change in FDG uptake between PET1 and PET2 (ΔSUV_max_) was significantly associated with the pCR rate (62% in the pCR group vs. 33% in the non-pCR group; p=0.008).

In summary, four markers were significantly associated with the pCR rate: tumor grade, PR expression, TP53 status and ΔSUV_max_. Best AUCs were observed with tumor grade (AUC = 0.88, 95%, CI: 0.83 – 0.93) and ΔSUV_max_ (AUC = 0.83, 95% CI: 0.63 – 0.97; Table 4). The predictive value of PR expression and TP53 status was more modest (AUC = 0.78 and 0.72, respectively, Table 4).

**Table 4:** Association between markers and pCR in the population of ER-positive, HER2-negative breast cancers

Variable	AUC	IC 95%
Tumor size	0.58	(0.37 – 0.78)
Histological grade	**0.88**	(0.83 – 0.93)
Progesterone receptor	**0.78**	(0.72 – 0.83)
Mutated P53	**0.72**	(0.50 – 0.90)
Ki 67 (IHC)	0.63	(0.40 – 0.83)
Ki 67 (Rt-qPCR)	0.63	(0.42 – 0.85)
CDC2	0.56	(0.30 – 0.81)
CDC20	0.62	(0.37 – 0.84)
KPNA2	0.68	(0.47 – 0.87)
MYBL2	0.47	(0.22 – 0.72)
GGI	0.61	(0.35 – 0.85)
SUV max tumoral PET1	0.62	(0.36 – 0.85)
SUV max tumoral PET2	0.61	(0.35 – 0.86)
Delta SUV max tumoral (%)	**0.83**	(0.63 – 0.97)

^*^95%CI = 95% confidence interval estimated using bootstrap.

AUC estimates with 95%CI excluding 0.50 are indicated in bold.

### Predictive markers of EFS

During a median follow-up of 57 months (17-196), 12 patients relapsed (2 local and 10 distant recurrences) among whom one patient died after relapsing. No association was observed between tumor characteristics and EFS, encompassing molecular biomarkers. The biological markers that were associated with the pCR (histological grade, PR expression and TP53 status) were not predictive of EFS in the Cox model (p = 0.93, 0.40 and 0.46, respectively). In this series of 75 luminal HER2-negative breast cancer patients, pCR was not significantly associated with EFS (p=0.34). However, only 6 patients (8%) reached pCR; none of these 6 patients relapsed, while 12 of the 69 patients with non-pCR tumors relapsed.

No association was observed between baseline SUV and EFS (p=0.60).

Finally, only ΔSUV_max_ (<12% vs. ≥12%) was significantly associated with patient’s outcome (p=0.047).

## DISCUSSION

The luminal breast cancers represent approximately two third of breast carcinoma as assessed by immunohistochemistry [24]. They are part of a heterogeneous spectrum of diseases, with different prognoses and different responses to treatment (Luminal A and B diseases) [2]. In this subgroup of relatively good but heterogeneous prognosis, a lot of tumors will not benefit from chemotherapy. We particularly need tools to predict chemotherapy response in neoadjuvant context.

In this work, we used the simplified classification of luminal breast carcinoma to define luminal A tumors that displays high level of ER and PR expression and low expression of proliferation (Ki67 <14%) and growth factor receptor, and luminal B tumors that displays low level of ER and PR expression or PR absent and higher expression of proliferation (Ki67≥14%) and growth factor receptor [25]. Furthermore, we considered only luminal B tumors without HER2 overexpression.

Ki67 is the most common marker used in clinical practice. Ki67 remains a subject of debate due to its cut-off values and also its inter-laboratory reproducibility. Automated scoring for Ki67 evaluation has been proposed to improve standardization and reproducibility [26]. Furthermore, it has been shown in breast cancers that high level of proliferation is predictive of pCR and is still a prognostic marker after NAC. However, Ki67 served as predictive marker of pCR remains controversial and is not applicable to all subtypes of breast carcinoma [11, 13, 27, 28]. In our study, we evaluated the predictive value of proliferation markers using either KI67 measured by IHC and with automated methods and also gene profiling analysis. We did not find any significant predictive value to this parameter. In a recent retrospective analysis of Ki67 expression in 77 breast cancer patients receiving NAC, Ingolf et al. showed no significant difference in Ki67 expression in responder’s *vs* non responders in the whole population. However, in the subgroup of 33 luminal breast carcinoma they showed significant difference in Ki67 expression in pCR *vs* no pCR samples (p=0.001) [29]. A recent meta-analysis evaluated the predictive value of KI67 in the NAC setting according to breast cancer subgroups. Eighteen out of 53 studies did not find any positive relationship between Ki67 and pCR. Five studies analyzed ER-positive breast cancers (excluding HER2+ tumors), among which only two showed that high Ki67 could predict pCR. [27].

We also evaluated the predictive value of the reduced GGI (GGIr), including a set of 4 genes (MYBL2, KPNA2, CDC2 and CDC20 which together cover all phases of the cell cycle as previously described [12]. In this context of luminal breast cancer, their expressions were not significantly different in pCR and no pCR tumors. In addition, none of them were predictive of EFS.

On the contrary, histological grade, PR expression and P53 status were predictive of pCR. Grade 3 tumor’s (100% in patients with pCR *vs.* 0 % in non-responders, p=0.0004), PR-negative BC (100% *vs.* 43%, p=0.010), and tumors with mutated TP53 (67% *vs.* 24%, p=0.042) better responded to chemotherapy. Others studies showed that the absence of PR expression was associated with worst outcome in luminal BC patients [30, 31]. As expected, in advanced breast cancers we observed a relatively high level of TP53 mutations (27%) in concordance with our previously published data in luminal A (17%) and luminal B (41%) BC [32] or globally in luminal BC (26%) [33]. In addition, Silwal-Pandt et al. analyzed a series of 1,420 breast cancer patients from the METABRIC cohort and showed that 9.3 % of luminal A and 24.5 % of luminal B tumors harbored mutated TP53 [34]. The molecular subtype was more precisely assessed using the PAM50 assay. We previously reported that TP53-mutated locally advanced breast cancers, mainly triple negative, had a higher rate of pCR to dose dense doxorubicin-cyclophosphamide chemotherapy [35, 36]. However, the association of TP53-mutated status with response to anthracyclins and taxanes is not clear [37]. Using a TP53 functional assay, we showed here that TP53 status may be a useful marker in ER-positive advanced tumors treated by EC-D chemotherapy regimen. Interestingly, a recent study based on a cohort of 115 ER-positive early breast cancers, showed that the TP53 mutation-associated gene expression signature was a powerful prognostic indicator for ER-positive tumors [38]. To date, it is now possible to extensively analyze the molecular P53 status by panel NGS for diagnosis. Furthermore, promising new developments should be considered looking at the first results of preclinical studies of mutant TP53 targeted therapy [39].

Metabolic markers, assessed by tumor ^18^FDG uptake, showed good predictors of pCR in patients treated with NAC, mainly in aggressive subtypes of breast cancer such as triple negative BC [6, 7, 10, 11, 14]. However, the role of FDG PET/CT is more uncertain in ER-positive / HER2-negative breast carcinoma. Before treatment, this subtype is associated with less intense ^18^FDG uptake than some other phenotypes such as triple negative breast cancer [40]. For this reason, it could be difficult to use the decrease in FDG uptake under treatment to assess treatment response [15].

However, we observed that the ΔSUVmax (change of FDG uptake after 2 cycles of chemotherapy) was predictive of pathological response, consistently with other studies [10, 41]. Interestingly, this parameter was the only biomarker associated with the event free survival. We did not observe any prognostic value of TP53 mutations, of PR-expression nor histological grade in our series of ER-positive patients. However, the follow-up period was limited, with a median time of 57 months (17-196). Many recurrences in patients with ER-positive / HER2-negative tumors occur between 5 and 10 years after treatment, or even later. Only ΔSUV_max_ (<12% vs. ≥12%) was significantly associated with patient’s outcome (p=0.047). The cut-off of 12% is low in comparison with others studies. This low value could be explained by the faint FDG uptake in luminal breast cancer [34] and a lower decrease of FDG tumor uptake under chemotherapy in comparison to more aggressive breast cancer subtypes such as triple negative or HER2-positive BC [10].

Contrarily to recent papers, we did not find that the absolute value of pre-treatment SUVmax was predictive of patient outcome [31, 42]. The limited number of patients in the present series might be responsible for a lack of power in the statistical analyses which could explain non-significant associations in the present study.

Our study has other limitations. We evaluated only the SUV parameter while other PET parameters showed potential interest in the luminal BC subgroup, notably parameters based on volume (metabolic tumor volume and total lesion glycolysis) [31, 42]. We also evaluated only the FDG uptake while other PET tracers could be of interest in luminal tumors, especially the 18F-Fluoroestradiol PET [43, 44].

In conclusion, our study confirms the prognostic value of tumor metabolic assessment, by the ΔSUVmax in ER-positive /HER2-negative advanced breast cancers treated in neoadjuvant context. Interestingly, we also observed a predictive value of P53 mutated status to predict pCR, in addition to histological grade, and PR expression in ER-positive breast cancers. These findings, if confirmed, could be helpful to select high-risk patients within trials investigating novel treatment strategies. However, only the variation of FDG uptake early during the NAC regimen (ΔSUVmax) was associated with patient’s survival. Further studies are needed to confirm our results in larger series of patients and a longer follow up.

## PATIENTS AND METHODS

### Patients, treatment and samples

The present study concerns a retrospective series of 75 HER2-negative luminal breast cancer patients with stage II-III breast cancer treated at Saint-Louis University Hospital (Paris, France) by neoadjuvant chemotherapy (NAC from July 2007 to August 2014). All patients were treated by a sequential regimen of 4 cycles of Epirubicin 75 mg/m^2^ plus Cyclophosphamide 750 mg/m^2^ followed by 4 courses of Docetaxel 100 mg/m^2^ (EC-D). All patients underwent breast-conserving surgery or mastectomy and axillary lymph node dissection after NAC. After surgery, patients received loco-regional radiation therapy (tailored to disease stage and breast surgery results) and adjuvant endocrine therapy for 5 years (Tamoxifen in premenopausal women or aromatase inhibitors in postmenopausal women).

Each patient had 2 PET/CT scans, one before the neoadjuvant chemotherapy (PET_1_) and another after 2 cycles (PET_2_). In the present retrospective analysis (NCT02600442), we only included consecutive patients with available frozen and fixed diagnostic biopsy, and with complete clinical and pathological data. Patients with synchronous distant metastases were not included.

The main objective of the present study was to validate early individual prediction of response to EC-D using a combination of FDG uptake (at baseline, after 2 cycles and its change) and proliferation evaluation on initial biopsy (Ki67 protein level by immunohistochemistry; Ki67 mRNA level and the mRNA expression of the most pertinent genes of the Genomic Grade Index [GGI] component by RT- qPCR), along with clinical and pathological characteristics, in patients with stage II/ III luminal breast carcinoma. A second objective was to evaluate the prognostic value of these parameters.

The Institutional Review Board approved the study and stated that no informed consent was needed, considering the non-interventional design of this analysis (n° IRB 00003835, French ethics committee Paris-Saint-Louis, n°2013-27NICB).

### Tumor histology and immunohistochemistry

Breast cancer and luminal subtype was proved on the initial core-needle biopsy. Histological grade was determined using the modified Scarff-Bloom-Richardson grading for invasive carcinoma. All biopsy specimens were fixed with 10% neutral phosphate-buffered formalin and paraffin-embedded. Four μm-thick slices of representative tumor blocks were stained with hematoxylin and eosin (H&E). Luminal breast tumors were defined on the basis of immuno-histochemical test results, using specific antibodies and an automated immunostainer (Ventana XT; Tucson, AZ, USA). Tumors were considered Estrogen Receptor (ER) and Progesterone Receptor (PR) positive if more than 10% of tumor cells expressed ER and/or PR immunostaining, referring to the routine clinical practice in France [18]. HER2 was analyzed by immunohistochemistry (IHC). Only tumors with no overexpression (overexpression defined as uniform HER2 immunostaining, intense membrane staining of >30% of invasive tumor cells (IHC 3+) and absence of HER2 amplification were kept.

The proliferation status was evaluated by immunohistochemistry of KI67, using MIB-1 antibody (dilution 1:100; Code M7240, Dako, Glostrup, Denmark). Ki-67 expression was assessed as a percentage of stained cells and analyzed both directly by the pathologist and automatically using the image analysis software Hamamatsu NDP Analyze. The threshold for Ki67 positivity was 14% stained cells whatever was the intensity of staining.

A simplified classification of these luminal tumors was defined using immunohistochemical characteristics: luminal A was defined as ER positive, PR positive, grade 1 or 2 and low proliferation status and luminal B phenotype was defined as ER positive ± PR positive, high grade and high proliferation status.

### RNA extraction and cDNA synthesis

Frozen sections of biopsies dedicated to RNA extraction were prepared by breast pathologists and were processed under RNAse-free conditions; RNA was extracted by phenol–chloroform method. First-strand cDNA synthesis was performed with 1μg total RNA using Superscript II Reverse Transcriptase (Invitrogen Corporation) in a final volume of 20 μL, as previously described [19].

### Real-time RT-qPCR analysis

Quantitative PCR analysis was performed on 10 ng cDNA in duplicate. A 5 μL diluted sample of cDNA was added to 20 μL of the PCR mix. The thermal cycling conditions comprised an initial denaturation step at 95°C for 10 min, 45 cycles at 95°C for 15 sec, and annealing temperature, either 60°C or 65°C depending on the target, for 1 min.

All PCR reactions were performed using the ABI Prism 7500 Sequence Detection System (Applied Biosystems Inc., Forster City, USA). The PCR Core reagent kit was used for systems with Taqman probes (Eurogentec, Liège, Belgium). Primers and fluorescent probes were designed from published sequences using Primer express software (Applied Biosystems Inc.). BLASTN searches against dbEST and nr (the non-redundant set of the GenBank sequence database) were performed to confirm the total gene specificity of the chosen nucleotide sequences and the absence of DNA polymorphisms. Primers and probes sequences for Ki67, and MYBL2, KPNA2, CDC2, CDC20 mRNA expression, as components of the GGI, are presented in Supplementary Table 1 of supplementary data. TATA Box binding protein (TBP) was used as endogenous reference genes. Target quantities were normalized to TBP mRNA expression.

Human breast luminal cancer cell lines T47D cDNA was used to generate 7 points standard curves for each gene. Target quantities were normalized to the reference genes and calibrated using the second point of each standard curve. Final results were expressed as N-fold differences in target gene expression relative to the reference genes and the calibrator and are expressed as:

$$2_{\text{target}}\left(\text{Ct calibrator }-\text{ Ct sample}\right)/{\text{E}}_{\text{reference gene}}\left(\text{Ct calibrator }-\text{ Ct sample}\right)\text{,}$$

where Ct is the cycle threshold.

No reverse-Transcription Controls (NTC) were included in any batch of samples.

### P53 assessment

RNA extracted from frozen biopsy was used to determine p53 gene functional status using a highly efficient yeast functional assay (FASAY: Functional Analysis of Separated Alleles in Yeast), which evaluates the transactivation activity of p53 on a p53-responsive promoter stably integrated in the yeast genome as described by Flaman et al. [20]. RNA was reverse transcripted in cDNA using Random Hexamer and Superscript II; p53 transcripts were amplified by polymerase chain reaction (PCR) (exon 4–10) and cotransfected with the Gap repair plasmid in yeast. In this assay, yeast colonies transformed with wild-type or mutated *TP53* sequences appear as white and large or red and small, respectively. p53 was considered non-functional when more than 10% of the yeast colonies were red. Analysis of the split version of the test was performed to confirm this result and to localize the defect in the 5′ or 3′ part of the gene.

When p53 was considered non-functional with FASAY, mutant yeast colonies were analyzed to identify the genetic defect by Sanger sequencing.

Finally, we classified the mutations into two groups using the International Agency for Research on Cancer (IARC) p53 database, according to their impact on the protein: presence of a modified p53 protein (p-MOD) and absence of a p53 protein (p-NO) [21].

### ^18^FDG-PET/CT Imaging

Before 18FDG-PET/CT imaging, patients fasted for 6 hours and blood glucose level had to be less than 7 mmol/L. ^18^FDG (5 MBq/kg) was administered and imaging (from mid-thigh level to the base of the skull with the arms raised) started almost 60 minutes later. The Gemini XL PET/CT scanner (Philips Medical systems) was used. CT data was acquired first (120 kV; 100 mAs; no contrast-enhancement). PET emission data was acquired in a 3-dimensional mode, with 2 min per bed position. The attenuation-corrected images were normalized for injected dose and body weight, and subsequently converted into Standardized Uptake Values (SUV), defined as: [tracer concentration (kBq/mL)] / [injected activity (kBq)/patient body weight (g)].

A 3D region of interest (3D-ROI) was drawn around the primary tumor only. When present, lymph nodes were not encompassing within the volume. Indeed, in a previous study [10], we observed that SUV value measured in axillary lymph nodes in addition to the measure within the primary tumor was not of added value to predict response to neoadjuvant chemotherapy in luminal breast cancer.

The change in SUV_max_ (maximum SUV value within the ROI) after two cycles of chemotherapy was expressed as ΔSUV_max_ (%) = 100 × (2^nd^ cycle SUV_max_ - baseline SUV_max_)/baseline SUV_max_.

### Pathologic response after NAC and patients follow up

Pathological complete response (pCR) was defined as no evidence of residual invasive cancer in breast tissues and lymph nodes [4].

During neoadjuvant chemotherapy, patients underwent clinical examination every two cycles. After surgery, patients had follow-up visits every 6 months for 5 years, then yearly. Event-free survival (EFS) was defined as the time period between the date of baseline clinical examination (or the date of surgery if considering the impact of pathological response on EFS) and the date of the first event or of the last follow-up. Events included local, regional, or distant recurrences or death.

### Statistical analysis

Clinico-pathological data (age, menopausal status, clinical tumor size and clinical nodal status, histological type, grade, presence of lymphovascular invasion, ER status, PR status and HER2 status) were analyzed. For descriptive analyses, categorical variables are presented with counts (percentages) and quantitative variables with median value (interquartile range). Correlations between continuous values were estimated using Spearman’s rank correlation coefficients, with their 95% confidence interval (CI) estimated by resampling (from 1000 bootstrapped replicates) [22].

Categorical variables were compared according to pCR using Fisher’s exact test and continuous variables compared using Wilcoxon’s rank sum test.

The performance of histoclinical features, molecular markers, PET parameters (SUV_max_ measured at PET_1_, at PET_2_ and ΔSUV_max_) for early prediction of non-pCR was also quantified using receiver operating characteristics (ROC) analyses. Areas under the ROC curves (AUC) were estimated with the trapezoidal rule. Their 95% confidence intervals (CI) were estimated using stratified bootstrap (as implemented in the pROC library on R statistical platform, with the number of pCR/non-pCR observations held constant across samples) [23].

We performed a univariate analysis using Cox proportional hazards models to identify prognostic factors for EFS. Age and PET parameters were entered as continuous variables. Molecular markers were entered as continuous and discontinuous variables.

All tests were two-sided and *P* values ≤0.05 were considered statistically significant. Statistical analyses were performed using R software (version 3.0.2) (https://cran.r-project.org/).

## SUPPLEMENTARY MATERIALS TABLES



## Abbreviations

CT: computed tomography
^18^FDG: ^18^F-fluorodesoxyglucose
EFS: event free survival
pCR: pathological complete response
NAC: neoadjuvant chemotherapy
PET: positron emission tomography
SUV: Standardized Uptake Values
