# Supplementary material for: 18FDG-PET/CT and molecular markers to predict response to neoadjuvant chemotherapy and outcome in HER2-negative advanced luminal breast cancers patients
Source: Oncotarget. 2018 Mar 27;9(23):16343–53. doi: 10.18632/oncotarget.24674 (PMC5893244; doi:10.18632/oncotarget.24674)
Supplement: Supplementary file 1 [file oncotarget-09-16343-s001.pdf]

^{18}FDG-PET/CT and molecular markers to predict response to neoadjuvant chemotherapy and outcome in HER2-negative advanced luminal breast cancers patients

SUPPLEMENTARY MATERIALS

Supplementary Table 1: Gene expression analysis: primers and MGB probes

Gene Name	Id	Forward Primer	Reverse Primer	Probe	Amplicon size (bp)	exon
KPNA2	NM_002266.2	CTCTAGGAAACATTGCAGGTGATG	AGTGGGTCAACTGCACCGTACT	AGTGTTCCGAGACTTGGTTA	73	5-6
MYBL2	NM_002466.2	GACAGCAGGACTGGAAGTTCCT	ATTCAAAACTCTCAGCCACCTGTA	TAACCGCACTGACCAGCAATGCCA	86	3-4
CDC2 (CDK1)	NM_001786.4		Hs04177802_m1		109	3-4
CDC20	NM_001255.2		0426680_mH		115	10-11
